# The complete chloroplast genome sequence of *Styrax hemsleyanus* (Styracaceae)

**DOI:** 10.1080/23802359.2020.1715861

**Published:** 2020-01-24

**Authors:** Xiaogang Xu, Lili Tong, Yabo Wang, Yaoqin Zhang, Chongli Xia

**Affiliations:** a College of Biology and Environment, Nanjing Forestry University, Nanjing, China;; b Co-Innovation Center for Sustainable Forestry in Southern China, Nanjing Forestry University, Nanjing, China;; c School of Horticulture and Landscape Architecture, Jinling Institute of Technology, Nanjing, China

**Keywords:** *Styrax hemsleyanus*, Styracaceae, chloroplast genome, phylogenomics

## Abstract

*Styrax hemsleyanus* is valued for its beauty and fragrance. Here, we characterized the complete chloroplast (cp) genome of *S. hemsleyanus* using next generation sequencing. The circular complete cp genome of *S. hemsleyanus* is 158,027 bp in length, containing a large single-copy (LSC) region of 87,641 bp, and a small single-copy (SSC) region of 18,290 bp. It comprises 133 genes, including 8 rRNA genes, 37 tRNAs genes, and 88 protein-coding genes. The GC content of *S. hemsleyanus* cp genome is 36.95%. The phylogenetic analysis suggests that *S. hemsleyanus* is a sister species to *Styrax odoratissimus* in Styracaceae.

*Styrax hemsleyanus* Diels. (Styracaceae), a beautiful tree with abundant white fragrant flowers blooming in late spring, is a precious ornamental tree for landscape greening. It is suitable for embellishing courtyards or planting on hillsides. But to date, there is still no complete cp genome was characterized for *S. hemsleyanus*, even for the *Styracaceae*. Here, we characterized the complete cp genome sequence of *S. hemsleyanus* (GeneBank accession number: MN 733525) based on Illumina pair-end sequencing to provide a valuable complete cp genomic resource.

Total genomic DNA was isolated from fresh leaves of *S. hemsleyanus* grown in Nanjing Forestry University campus in Nanjing, Jiangsu, China. The voucher specimen was deposited at the herbarium of Nanjing Forestry University (accession number NF2019368). The whole genome sequencing was carried out on Illumina Hiseq platform by Nanjing Genepioneer Biotechnology Inc. (Nanjing, China). The original reading was filtered by CLC Genomics Workbench v9, and the clean reading was assembled into chloroplast genome with SPAdes (Bankevich et al. 2012). Finally, CpGAVAS (Liu et al. 2012) was used to annotate the gene structure and OGDRAW (Lohse et al. 2013) was used to generate the physical map. Based on the maximum likelihood (ML), the phylogenetic tree was deduced by MAFFT (Katoh and Standley 2013).

The circular genome of *S. hemsleyanus* was 158,027 bp in size and contained two inverted repeat (IRa and IRb) regions of 26,048 bp, which were separated by a large single copy (LSC) region of 87,641 bp, and a small single copy (SSC) region of 18,290 bp. A total of 133 genes are encoded, including 88 protein-coding genes (81 PCG species), 37 tRNAs gene (30 tRNA species), and eight rRNA genes (four rRNA species). Most of genes occurred in a single copy; however, 7 protein-coding genes (*ndhB, rpl2, rpl23, rps12, rps7, ycf2 and ycf15*), 7 tRNA genes (*trnA-UGC, trnI-CAU, trnI-GAU, trnL-CAA, trnN-GUU, trnR-ACG and trnV-GAC*), and 4 rRNA genes (*4.5S, 5S, 16S, and 23S*) are totally duplicated. A total of nine protein-coding genes (*atpF, ndhA, ndhB, petB, petD, rpl16, rpoC1, rps16 and rpl2*) contained one intron while the other 3 genes (*clpP, ycf3, rps12*) had two intron each. The overall GC content of *S. hemsleyanus* genome is 36.95%, and the corresponding values in LSC, SSC and IR regions are 34.79%, 30.25%, and 42.93%, respectively.

The phylogenetic analysis was conducted based on 27 Styracaceae cp genomes and 3 taxa (Symplocaceae, Actinidiaceae, and Theaceae) as outgroups with sequenced cp genomes. We found that *S. hemsleyanus* was clustered with other families of Ericales such as Symplocaceae, Actinidiaceae, Theaceae, with 100% boot-strap values (Figure 1). In addition, *S. hemsleyanus* was highly supported to be a sister species to *Styrax odoratissimus* in Styracaceae. Furthermore, this study will pave the way for future research to understand the genomic information of the chloroplasts of the Styracaceae and this chloroplast resource could also be utilized on the phylogeny, DNA barcoding, and conservation genetics.

**Figure 1.**
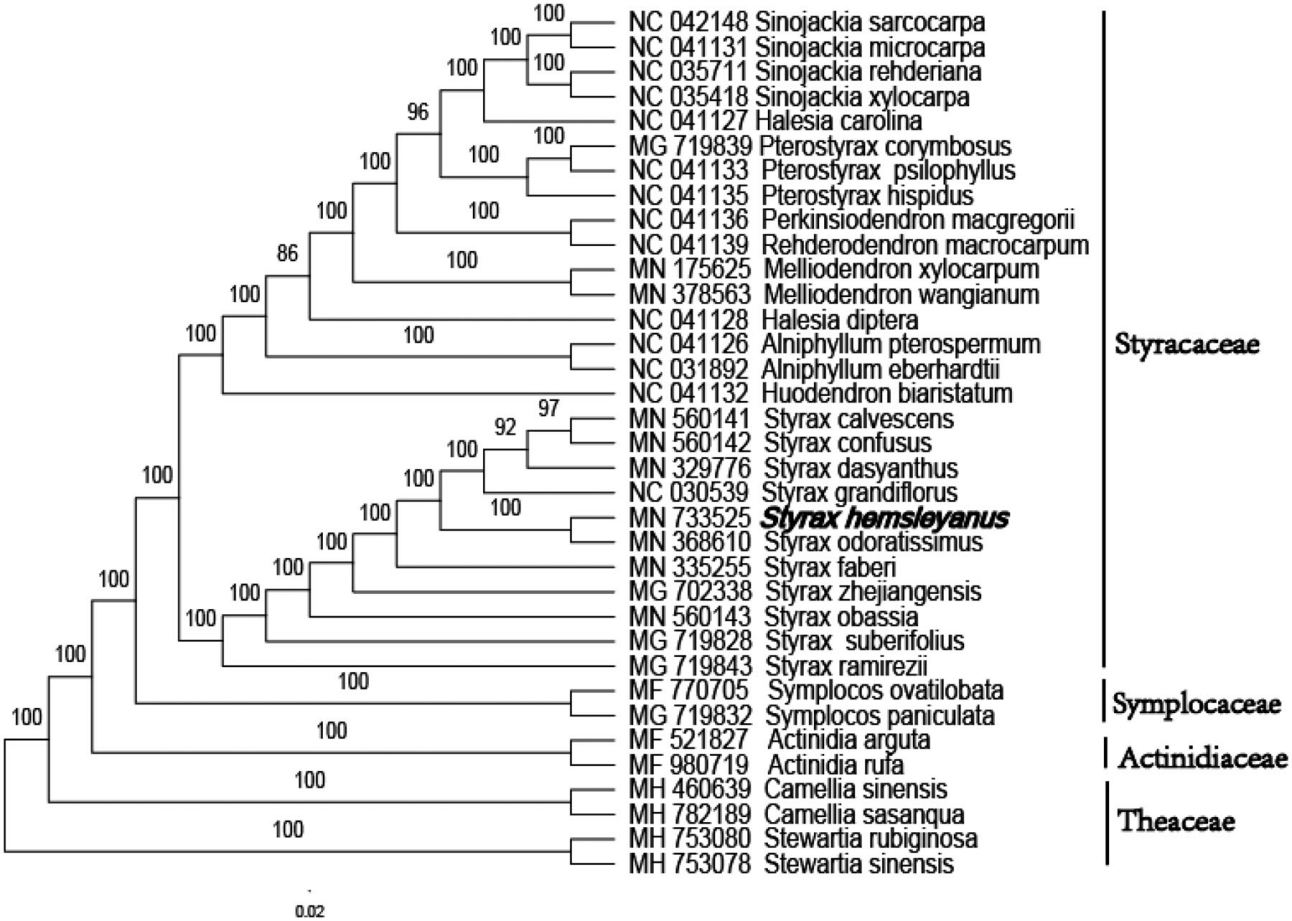
Phylogenetic tree inferred by maximum-likelihood (ML) method based on the complete chloroplast genome of 35 representative species. The bootstrap support values are shown at the branches.
